# Using Residence Time Distributions (RTDs) to Address the Traceability of Raw Materials in Continuous Pharmaceutical Manufacturing

**DOI:** 10.1007/s12247-015-9238-1

**Published:** 2015-11-14

**Authors:** William Engisch, Fernando Muzzio

**Affiliations:** Department of Chemical and Biochemical Engineering, Rutgers University, 98 Brett Rd., Piscataway, NJ 08854 USA

**Keywords:** Continuous Processing, Residence time distribution, Traceability, Batch definition, Process analytical technology (PAT)

## Abstract

Continuous processing in pharmaceutical manufacturing is a relatively new approach that has generated significant attention. While it has been used for decades in other industries, showing significant advantages, the pharmaceutical industry has been slow in its adoption of continuous processing, primarily due to regulatory uncertainty. This paper aims to help address these concerns by introducing methods for batch definition, raw material traceability, and sensor frequency determination. All of the methods are based on established engineering and mathematical principles, especially the residence time distribution (RTD). This paper introduces a risk-based approach to address content uniformity challenges of continuous manufacturing. All of the detailed methods are discussed using a direct compaction manufacturing line as the main example, but the techniques can easily be applied to other continuous manufacturing methods such as wet and dry granulation, hot melt extrusion, capsule filling, etc.

## Introduction

Pharmaceutical manufacturing has a long history of developing and manufacturing drug product in batches. This production technique was used for industrial chemicals and other consumer products long before the industrial revolution (eighteenth century) when an initial shift from batch to continuous processing occurred. Due to continuous process advantages, today, the majority of commodity chemicals, petrochemicals, food, and consumer products are manufactured continuously, leaving behind pharmaceuticals, which are still made with traditional batch processes. Many sources have suggested that pharmaceutical manufacturing has been frozen in time due to regulatory requirements that generate large amounts of paperwork, causing huge monetary cost in production delays resulting from even minor manufacturing changes (see, for example, a *Wall Street Journal* article on this topic [1]). This has lead to fearful, conservative cultures within the industry, which would rather remain steadfast with old and familiar technology rather than evolve with new technologies that improve the industry.

With the goal of modernizing and spurring technological improvement in the regulation of pharmaceutical manufacturing and product quality, in August 2002, the Food and Drug Administration (FDA, http://www.fda.gov) launched a regulatory modernization initiative, meant to encourage early adoption of new technological advances, facilitate industry application of modern quality management techniques, encourage implementation of risk-based approaches, ensure regulatory policies are based on state-of-the-art science, and enhance the consistency and coordination of drug quality regulatory programs. [2] A series of guidances have since been published, which further encourage significant changes to processes used to manufacture pharmaceuticals. The FDA has published the initial process analytical technology (PAT) framework [3], which supports the move from static batch processing to more dynamic approaches that mitigate the risk of producing poor-quality product. The International Conference on Harmonization (ICH, http://www.ich.org) implemented a trio of quality guidances: Q8(R2), Q9, and Q10 [4]–[5], which introduced valuable new concepts such as quality by design (QbD).

Although the regulatory guidances describe in detail what is necessary, they provide little explanation about how to accomplish it. To begin filling this gap, the International Society for Pharmaceutical Engineering (ISPE, http://www.ispe.org) launched the Product Quality Lifecycle Implementation (PQLI) initiative in 2007. This initiative aims to provide practical solutions for implementation challenges of the ICH guidances [6–8], while still recognizing that there are multiple satisfactory ways to address the concepts described in the guidelines [6]. However, there is little focus on providing solutions that directly apply to continuous processing.

One of the main approaches to modernizing and improving pharmaceutical manufacturing is continuous processing, which in recent years has gained attention of both the industry and regulatory authorities [9–17]. Continuous manufacturing approaches have many advantages over traditional batch methods, which have motivated many other industries to adopt them [11, 18]. Continuous processing equipment has a much smaller footprint leading to lower equipment costs. Because all the processing steps are interconnected, no intermediate storage is needed, lowering the necessary material inventory. Unlike batch processing, the smaller scale and ability to process different amounts of material simply by changing the production time make continuous systems versatile in both the clinical and commercial scales without the need for scale-up.

Continuous systems with automation and process control result in high-quality (low-variability) products, whereas batch processing is far less understood, resulting in often unpredictable product quality [11]. Blend segregation has been shown to be prominent in batch systems, while continuous systems have demonstrated the ability to process segregating mixtures without issue [19]. Moreover, a properly designed continuous system handles small portions of material at any given moment, increasing material monitoring scrutiny. This is unfeasible for large-scale batch processes with a similar throughput. Utilizing product and process understanding with properly implemented online PAT, continuous manufacturing readily fits the criteria needed to enable real-time release testing (RTRt), leading to rapid and reliable batch release of high-quality product. In spite of these vast advantages, continuous manufacturing also has significant challenges, and if implemented incorrectly, continuous processes will fail.

Two notable challenges are batch definition and raw material traceability, both required by regulation. [20] This work presents a method based on the residence time distribution (RTD), which can be used to address both of these challenges. The RTD is also used to examine the sensing frequency, with the goal of defining a sensing speed that would ensure that any unacceptable content uniformity variations would be detected and handled. As a case study, a simplified quality risk management process, including assessment and control, was completed for a direct compression case study, which identified high-risk content uniformity issues and reduced them through redesign that improved system robustness.

In the chemical processing field, the residence time distribution (RTD) is used to describe how a material travels inside the unit operations of a continuous process system. RTD is a critical, yet underutilized tool in pharmaceutical process understanding, quality assurance, and equipment and sensing design. Although traditionally applied to fluid systems [21], there have been many publications showing this the same probability-based time distribution also applies to granular or powder systems. [22–30]

## Continuous Manufacturing System

**Fig. 1:**
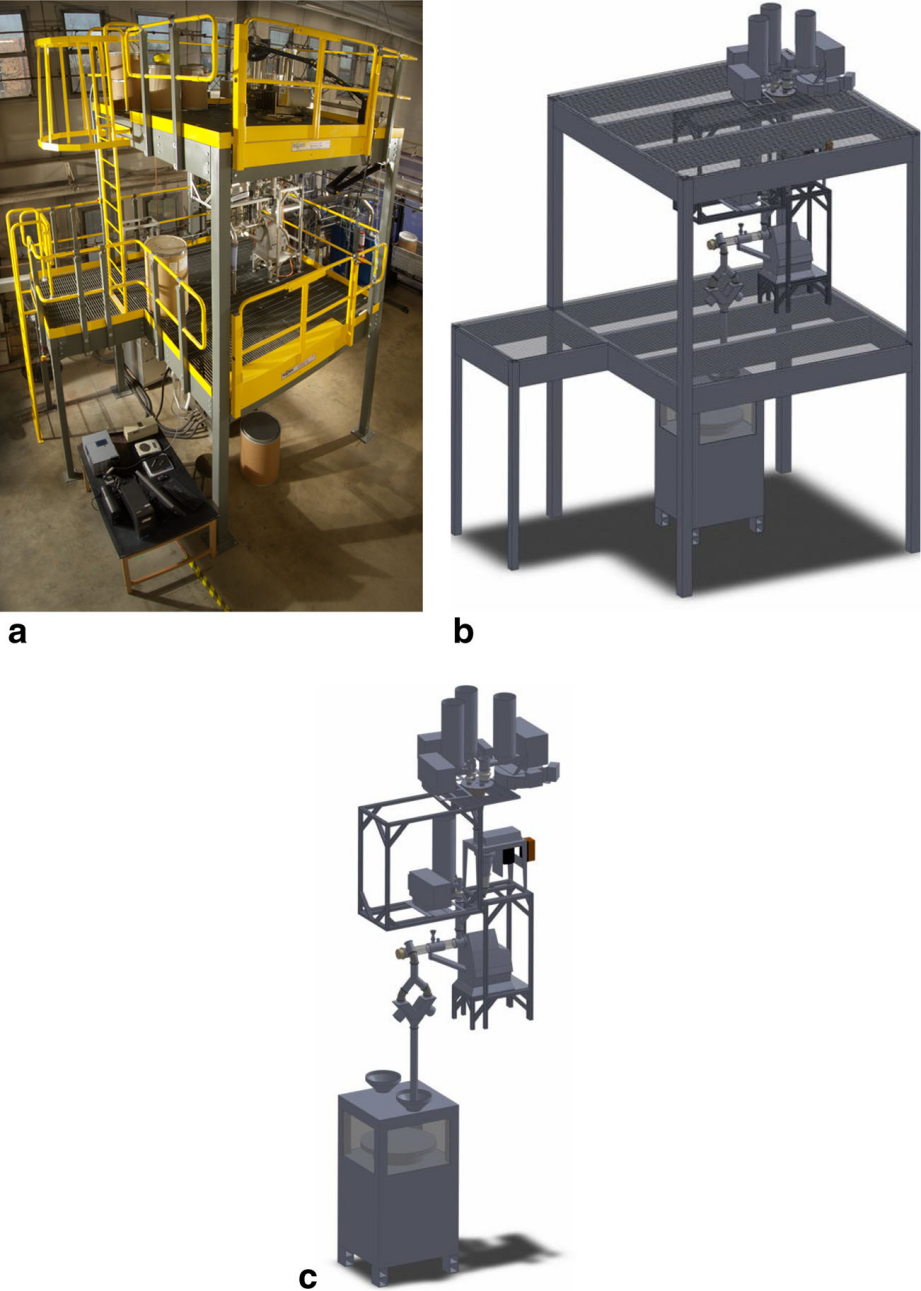
ERC-SOPS prototype direct compaction line located at Rutgers University: **a** Photo of the platform. **b** Model of the platform. **c** Simplified model of the system showing the connected unit operations without the scaffolding

The model system used for the methods developed in this work is the prototype continuous direct compaction (DC) manufacturing system, which was developed and built by the Engineering Research Center for Structured Organic Particulate Systems (ERC-SOPS, http://www.ercforsops.org/) located at Rutgers University. A photo and model of the continuous manufacturing platform are shown in Fig. 1a, b, and a simplified model highlighting the unit operations is shown in Fig. 1c. The continuous DC system was constructed on a three-tiered scaffolding platform, which has multiple loss-in-weight feeders on the highest level. The feeders supply the multiple components of formulation through to a Quadro Comil, which is located on the middle level and serves a triple purpose. The Comil sieves breaking large agglomerates, performs initial high shear mixing, and ensures intimate contact of poorly flowing ingredients with glidants, thus improving blend flow properties. Also, on the middle level, the Comil’s exit passes milled material to a Glatt continuous mixer, which consists of a horizontally rotating shaft with triangular-shaped paddles that mix the blend as it travels through the tubular body. An additional feeder supplies lubricants (i.e., MgSt) directly to the blender, bypassing the Comil. Following the mixer is a Kikusui tablet press, which compresses the blended formulation into tablets at the ground floor level.

## Methods

### Residence Time Distribution Experiments

The residence time distribution (RTD) can be easily obtained for all unit operations in a continuous line with a tracer response experiment performed for each unit operation separately and for the mechanically integrated line as well. In this testing, a pulse or step change of tracer is added to the inlet of the continuous equipment being characterized, and the response of the tracer concentration profile at the outlet is measured. The concentration measurements can be recorded using online spectroscopy, or samples can be collected for off-line measurement. In either case, it is important that the tracer concentration be readily measureable by an analytical technique. Additionally, the presence of the tracer should not impact the flow properties of the bulk material for which the RTD measurements are being taken, because the RTD is highly dependent on the flow behavior of the material within the apparatus. Any significant changes to the flow behavior will cause the measured RTD not to be representative of the material.

Furthermore, the RTD can be sensitive to all process parameters, which means that the entire design space of a unit operation needs to be investigated. This is particularly important, because a continuous system with process control will change process parameters to maintain a consistent product.

For tracer pulse tests, the response will be a concentration profile, *C*(*t*), that has the same shape as the residence time distribution, *E*(*t*). The RTD can be calculated by normalizing the concentration profile by the area underneath the profile:

1 $$ E(t)=\frac{C(t)}{{\displaystyle \underset{0}{\overset{\infty }{\int }}C(t)dt}} $$

It is important that the data set for the concentration profile be completed and includes the entire tail. If the profile is not complete or the tail is very long, the RTD will be inaccurate. If this occurs, it is possible to extrapolate the tail as an exponential decay, which will improve accuracy of an incomplete dataset [31].

The tracer pulse technique also relies on the ability to add a pulse that is as close to instantaneous as possible. If this is not possible or the residence time is very short, this can also add inaccuracies. However, when correctly applied, this method is the most direct method for determining the RTD [31].

If the pulse technique is not reliable, an alternative is the step change technique. For tracer step change tests, the response will be a concentration profile with the same shape as the cumulative distribution function (CDF), *F*(*t*). To calculate the CDF, the concentration profile needs to be normalized so that the initial value is 0 and the final value is 1:

2 $$ F(t)=\frac{C(t)-{C}_{\mathrm{initial}}}{C_{\mathrm{final}}} $$

where *C*_initial_ and *C*_final_ are the initial and final tracer concentrations. Typically, the initial tracer concentration would be 0, which simplifies this equation to:

3 $$ F(t)=\frac{C(t)}{C_{\mathrm{tracer}}} $$

The cumulative distribution function (step response) and residence time distribution (pulse or point response) are related by the following equations:

4 $$ F(t)={\displaystyle \underset{0}{\overset{t}{\int }}E(t)}\kern0.5em dt $$

5 $$ E(t)=\frac{dF(t)}{dt} $$

A residence time distribution has several moments that can be used to characterize its shape. For this study, only the first two integer centered moments are used, respectively the mean residence time and the variance (square of standard deviation). The equations for the mean residence time and the variance are as follows:

6 $$ \tau ={\displaystyle \underset{0}{\overset{\infty }{\int }}tE(t)dt} $$

7 $$ {\sigma}^2={\displaystyle \underset{0}{\overset{\infty }{\int }}{\left(t-\tau \right)}^2E(t)dt} $$

The mean residence time can be used to quantify the center of the residence time distribution, whereas the standard deviation is used for determining its width. These moment values are useful for describing the shape of a distribution without relying on the entire distribution.

### Residence Time Distribution Fitting

Continuous unit operations vary dramatically in both function and geometry, and correspondingly, the residence time distribution (RTD) of any unit operation is equally as varied. In liquid flow and mixing applications, this has resulted in the development of many RTD models, some of which may not be appropriate for solid unit operations.

**Fig. 2:**
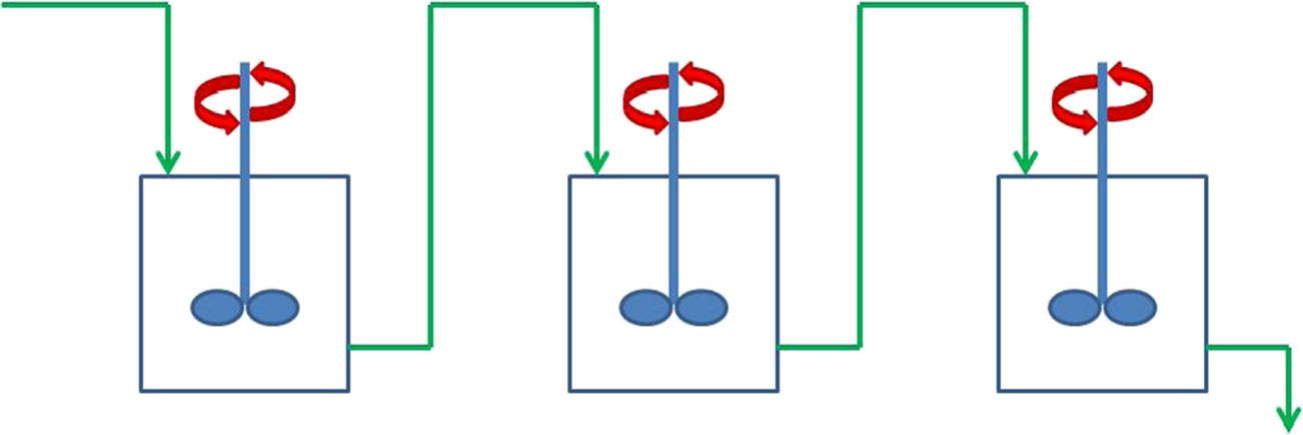
Depiction of the tanks-in-series model where *n* = 3

**Fig. 3:**
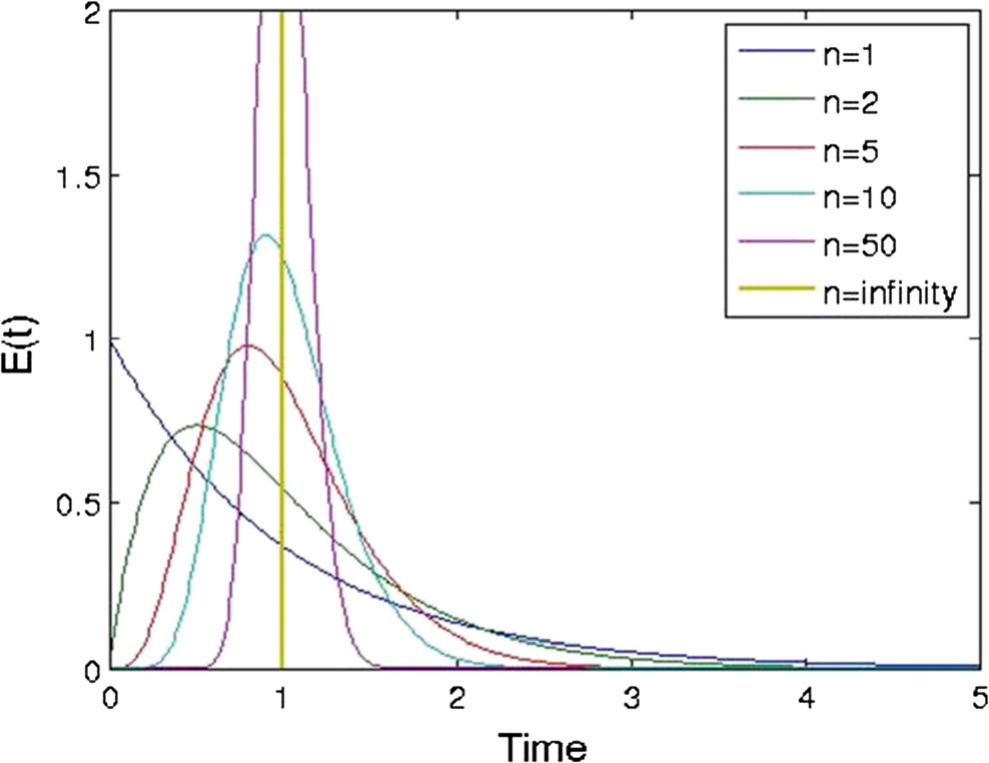
Residence time distributions for tanks-in-series model having a mean residence time of 1 and a number of tanks ranging from 1 to infinity

However, the examples shown in this work use the “stirred tanks in series” model, which is an empirical model based on equally sized continuously stirred tank reactors (CSTRs) placed in series (see Fig. 2). The model for a CSTR assumes a mixed vessel with perfect back-mixing. However, placing CSTRs in series results in a model for realistic mixing. Figure 3 shows a range of residence time distributions modeled with tanks in series. The number of tanks in this figure ranges from 1 up to infinity. A larger number of tanks in series result in a narrower distribution. An infinite number of CSTRs in series are equivalent to a plug flow tubular reactor (PFR), which does not have any axial mixing and is represented by a pulse response.

Generalizing the model for tanks in series results in the following equations for RTD [31]:

8 $$ E(t)=\frac{t^{n-1}}{\left(n-1\right)!{\left(\frac{\tau }{n}\right)}^n}{e}^{\left(\frac{- nt}{\tau}\right)} $$

where *τ* is the mean residence time and *n* is the number of CSTRs. The concentration profile for the pulse response testing is similarly generalized by:

9 $$ C(t)={C}_0E(t)={C}_0\frac{t^{n-1}}{\left(n-1\right)!{\left(\frac{\tau }{n}\right)}^n}{e}^{\left(\frac{- nt}{\tau}\right)} $$

where *C*_0_ depends on the amount of material added in the pulse.

The RTD experimental data was fit to the tanks-in-series model using a built-in Matlab function, “lsqcurvefit,” which is a least squares curve fitting function based on the trust-region-reflective algorithm described by Coleman et al. [32, 33]. The concentration profile defining parameters (*C*_0_, *τ*, and *n*) are determined by this least squares technique, which seeks these values while minimizing the sum of square (SS) error between estimated and experimental values:

10 $$ \mathrm{S}\mathrm{S}=\underset{X}{ \min }{{\displaystyle \sum_i\left(C\left(X,{t}_i\right)-{C}_i\right)}}^2 $$

where *C*(*X*,*t*_*i*_) is the estimated concentration, *t*_*i*_ and *C*_*i*_ represent the *i*th points from the experimentally collected time and concentration datasets, and *X* is the parameter set for the model:

11 $$ X=\left[{C}_0,\tau, n\right] $$

### Convolution

A single residence time distribution can be used to trace the passage of materials through a continuous flow system. Since the RTD is the pulse or point response of the system, if the system response is linear (i.e., if the tracer does not modify the flow properties of the blend), any point in time will behave and spread through the system just like a pulse of equal magnitude. A measured input stream could be represented with a string of discrete values representing the fluctuations in the stream. Using the convolution integral for mixing:

12 $$ {C}_{\mathrm{out}}(t)={\displaystyle \underset{0}{\overset{t}{\int }}{C}_{\mathrm{in}}\left(t-t\hbox{'}\right)}E\left(t\hbox{'}\right)dt\hbox{'}={\displaystyle \underset{0}{\overset{t}{\int }}{C}_{\mathrm{in}}}\left(t\hbox{'}\right)E\left(t-t\hbox{'}\right)dt\hbox{'} $$

represented in short hand by the convolution operator equation:

13 $$ {C}_{\mathrm{out}}(t)={C}_{\mathrm{in}}(t)*E(t) $$

it is possible to predict the outlet of a unit operation as long as the concentration of the inlet stream, *C*_in_(*t*), and the RTD, *E*(*t*), are both known. This can be extended to a series of unit operations by calculating the overall RTD recursively, for example, for two unit processes, as:

14 $$ E(t)={E}_1(t)*{E}_2(t) $$

where *E*_1_(*t*) is the RTD from a first unit operation and *E*_2_(*t*) is from a second operation.

**Fig. 4:**
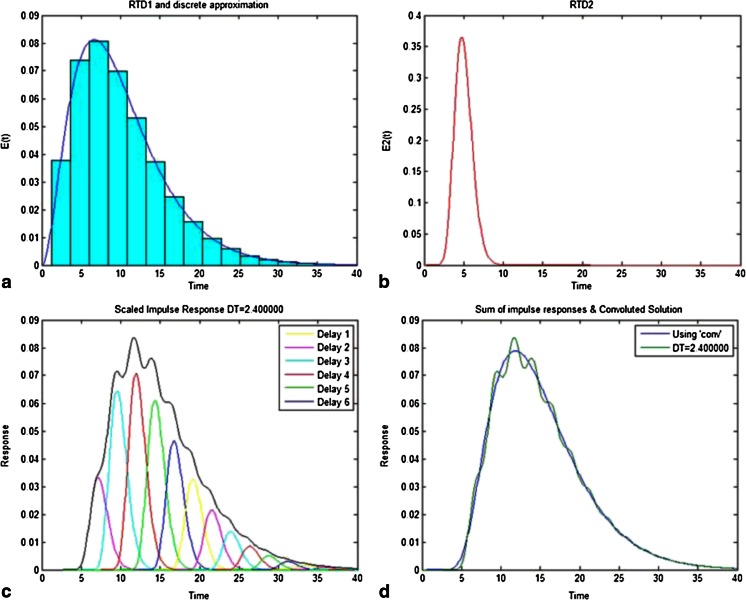
Visual representation of the convolution technique for two residence time distributions (RTDs), E1 and E2. **a** Discrete approximation of E1. **b** E2. **c** E1’s discrete approximation-scaled responses of E2 and their sum. **d** Sum of impulse responses for a time interval of 2.4 s and result from convolution function

**Fig. 5:**
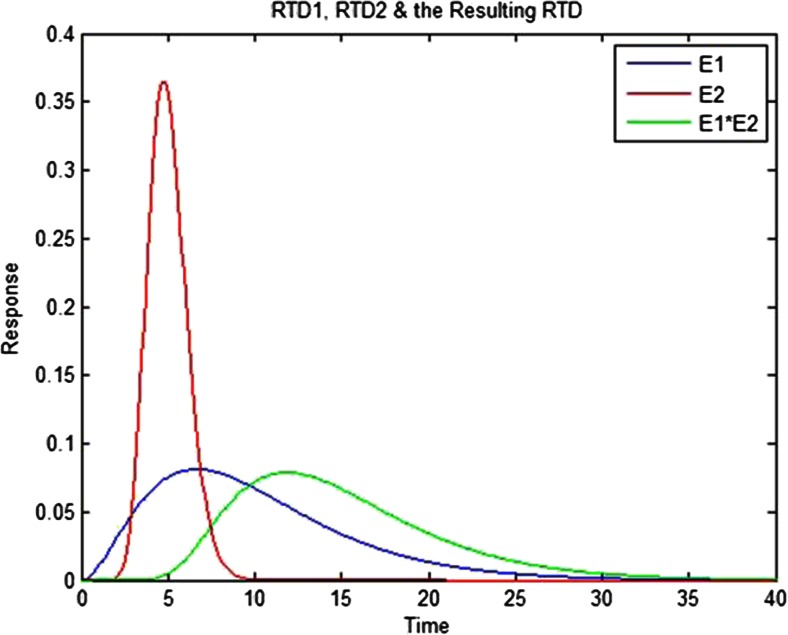
Representation of the convolution of two residence time distributions (RTDs), E1*E2, plotted with the two component RTDs, E1 and E2

This convolution technique is depicted in Figs. 4 and 5. In Fig. 4a, the first RTD, *E*_1_(*t*), is discretized with approximations for the time interval of 2.4 s, where the discrete version of the RTD is now represented by a sequence of bars. Figure 4b shows the second RTD, *E*_2_(*t*), which is scaled for each of the elements in the discrete approximation from Fig. 4a and is plotted in Fig. 4c. For example, the first element is 0 when *t* = 0, which is why the peak of *E*_2_(*t*), 0.36 at *t* = 5 s, results in the scaled response of 0 at 5 s. The second element, which is 0.0378 at *t* = 2.4 s, results in a product of 0.033 (0.36*0.0378*2.4), which is the value shown for the peak of the scaled response at *t* = 7.4 (5 s + 2.4 s). This was repeated for all of the elements in the discrete approximation, while the time was offset by 2.4 s for each subsequent approximation, which was the time interval. These are then summed, and are shown in Fig. 4d overlaid with the solution from the Matlab “conv” function. The “conv” function uses a time interval corresponding to the resolution of the RTD, which creates a smooth solution in contrast to the example, which was limited to the 2.4-s time interval. Figure 5 shows a plot of the two unit operation RTDs, *E*_1_(*t*) and *E*_2_(*t*), with their convoluted solution or overall RTD, which is both broader and has a longer mean residence time.

The Matlab function’s generalized definition is:

15 $$ E\left({t}_k\right)={\displaystyle \sum_j{E}_1\left({t}_j\right)}{E}_2\left({t}_k-{t}_j+\varDelta T\right)\varDelta T $$

where ∆*T* is the time interval for the two RTDs and *t*_*k*_ and *t*_*j*_ are the *k*th and *j*th points of the time array.

### Traceability of Raw Materials in Continuous Processing Systems

**Fig. 6:**
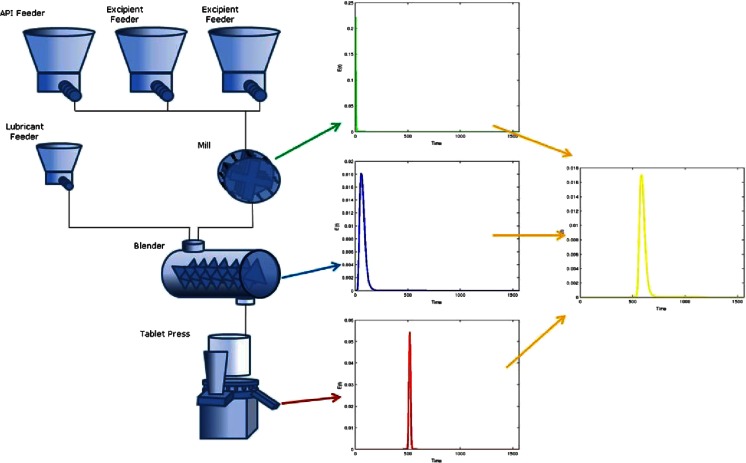
Residence time distribution of the individual unit operations and overall system

The overall process RTD can be determined using the mathematical tool of convolution in combination with the residence time distributions (RTDs) for each unit operation. Figure 6 shows a process flow diagram for a direct compaction continuous manufacturing system. After the feeders at the top, the first unit operation is a mill, which has a short and narrow RTD. Next is the continuous blender, which has significant back-mixing and therefore a broader residence time distribution. Finally is the tablet press, which has an even longer residence time due to the feed hopper and the feed frame, but only a small amount of back-mixing in the feed frame. Combining these three unit operations through the convolution technique yields an overall system RTD, which is both longer and wider than any of the individual unit operations. This overall system RTD can be used to trace raw materials across the entire system, all the way to the tablets.

RTD modeling of the system allows for tracking the evolution of any process disturbance through the process so that the affected downstream material can be easily identified as well as backtracking to pinpoint the source of the disturbance making it a useful predictive tool for risk management. However, RTD modeling needs to be utilized with other tools to be effective. For example, the ability to detect a disturbance is contingent of having appropriate sensors in optimal locations. Paired with an exceptional event management framework as described by Hamdan et al. [34], RTD modeling can provide the mapping needed for corrective action needed for exceptional events in the form of dynamic process changes or removal of out of specification material. The result is reduced variability and an improvement in product quality.

For simplicity of this depiction in Fig. 6, the RTD of the feeders and feeder refill system is not shown, but to trace raw material back to a drum will require mapping those unit operations as well. The method for this or other continuous systems is the same. The RTD for each feeder will be unique to the equipment and powder used under the actual operation conditions used. Because of this, each component will have a separate overall residence time distribution. This would be the case anytime multiple streams are combined. For example, consider a process to create a bi-layer tablet. The process would involve separate blending of the blend used to make each side of the tablet, usually in unequal proportion and having a different composition (i.e., a different active pharmaceutical ingredient (API)) causing the ingredients in the two sides to have different RTDs. However, RTDs vary monotonically with respect to material properties and processing conditions; thus, the development for predictive correlations for RTDs is entirely feasible [35].

## “Batch” Definition

One of the early barriers to developing and implementing continuous processing was, and to some extent remains, uncertainty regarding regulatory compliance. One of the main concerns is the ability to trace materials by batch and lot, a regulatory requirement. According to 21 CFR 210 [36], the definitions of batch and lot are:

Batch: “A specific quantity of a drug or other material that is intended to have uniform character and quality, within specified limits, and is produced according to a single manufacturing order during the same cycle of manufacture.”
Lot: “a batch, or specific identified portion of a batch, having uniform character and quality within specified limits’ or, in the case of a drug product produced by continuous process, it is a specific identified amount produced in a unit of time or quantity in a manner that assures its having uniform character and quality within specified limits.”

The regulatory definition of batch has no stipulation or requirement as to the method of manufacture, and in fact, the definition of lot specifically includes continuous processing. It is still necessary to define batch and lot to comply with various aspects of current good manufacturing practice [20, 37]. Compliance requires:

- Batch production and control records
- Laboratory conformance testing and release
- Investigation of failures or discrepancies
- Recall procedures

While both batch and lot are defined, precise specification of each is left to the manufacturer’s discretion and design. For a continuous manufacturing process, specification may be based on production time period, amount of material, variation in production, or maintenance cycles. A variation in production, such as a change in feedstock lot, may be the most appropriate method as a batch is “intended to have uniform character and quality” [36].

**Fig. 7:**
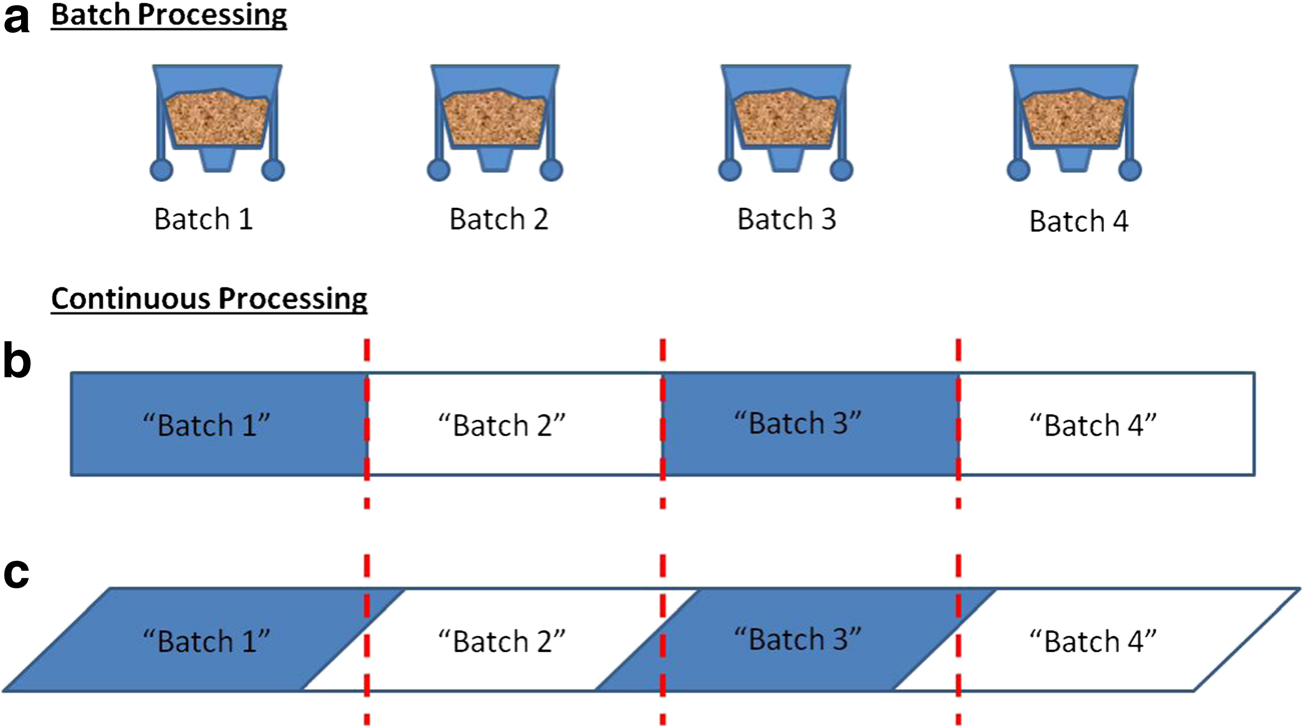
Visual comparison of batch definition for **a** “traditional” batch processing, **b** continuous “plug flow” processing, and **c** realistic (non-plug flow) continuous processing. The *dotted lines* represent arbitrary divisions between batches

In a batch process, the “batches” are physically separated into enclosed vessels, making batch identification straightforward (see Fig. 7a). In continuous manufacturing, a physically separated “batch” does not exist; instead, a continuous non-stop stream of product is generated. The lack of a physical barrier between batches in a continuous process causes the boundaries between batches to become confounded because of back-mixing across the system. A naive and unrealistic view of batch specification for a continuous processing might assume that there is no back-mixing. However, this is only true for an ideal plug flow system (see Fig. 7b), in which an arbitrary boundary would suffice and then the identification would be similar to that of batch processing. Such a plug flow system, however, would have no back-mixing capabilities and therefore would be unable to eliminate any variability entering the system due to either material properties or processing conditions. Thus, substantial back-mixing would be an intrinsic characteristic of any robust and effective continuous manufacturing process, and batch definition must address its presence.

Therefore, in a realistic continuous system (see Fig. 7c), which would have some amount of back-mixing, materials would comingle between subsequent batches. Although there is no specific regulatory conformance problem with using an arbitrary division, it must be determined how many batches are affected by any potential manufacturing inconsistency. Additional procedures would need to be developed to address these inconsistencies. See Fig. 7c for an example. In this case, if there were a need to recall “Batch 3,” then, it must be assumed that the recall might also apply to “Batch 2” and “Batch 4.” As the batches may be quite large, this would result in a large amount of recalled or rejected material. To solve this problem, smaller batches could be used, resulting in less material loss, but increased release-related testing (thus emphasizing the importance of RTRt). With any batch size, experimental qualification of the equipment must be determined to properly identify the batches that should be considered adulterated.

**Fig. 8:**
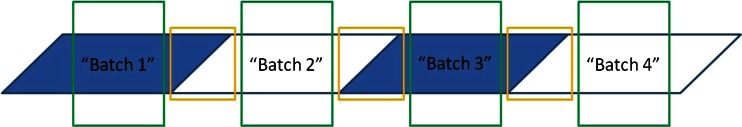
Depiction of batch definition for continuous processing, which removes the interface regions (in *yellow boxes*) between batches. The remaining material between these regions then become the batches (in *green boxes*)

An alternative to drawing an arbitrary line between batches in a continuous system would be to separate the interface region between batches and define the batch as the material between the interfaces. See Fig. 8. In the case where batches are specified by a component lot change, this method would ensure that each batch contains only a single feedstock lot. Removing the interface is analogous to the removal of the first and last parts of a batch made by batch processing, which is often performed to maintain uniform quality. However, the need to do this in batch processing is due to actual quality problems, such as blend segregation. In continuous manufacturing, such quality problems are minimized; thus, the possible need to discard the interface is entirely a regulatory compliance issue.

**Fig. 9:**
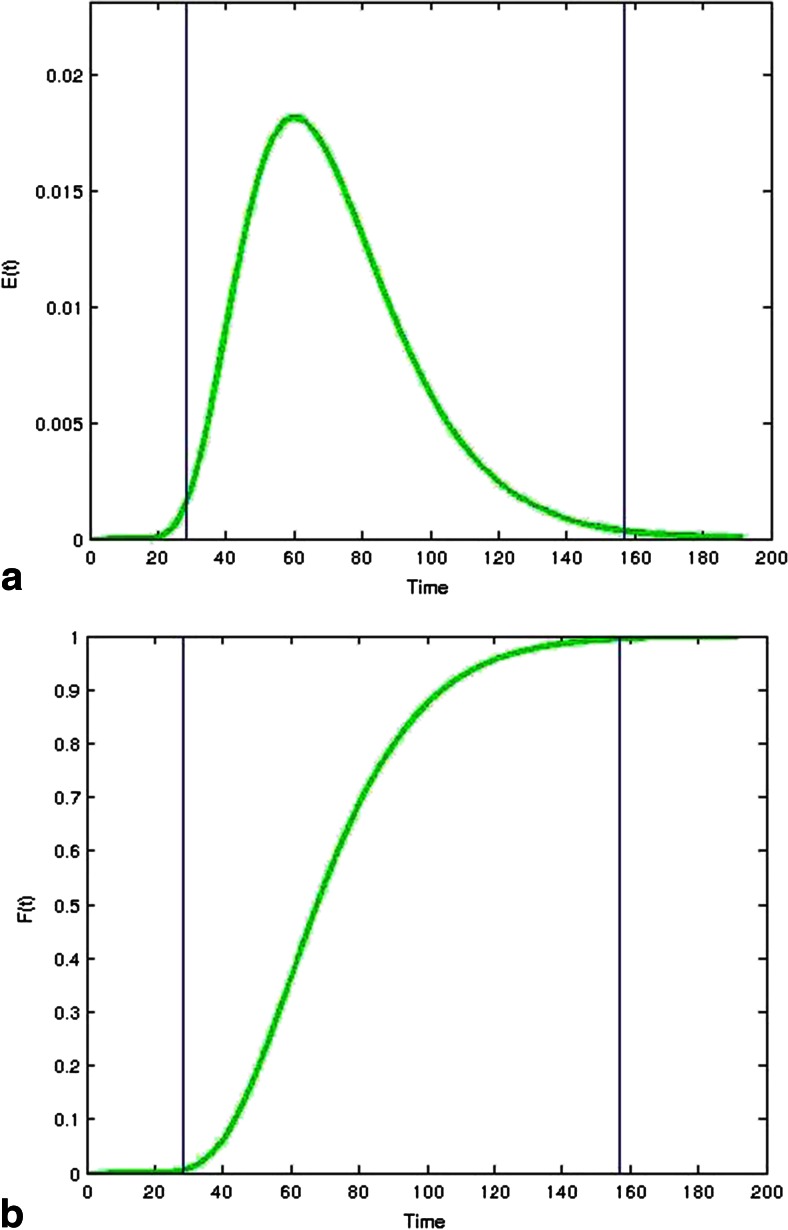
Define the boundaries of a batch for a continuous process by using **a** residence time distribution (RTD) and **b** cumulative distribution function (CDF). The boundaries shown here are 0.5 and 99.5 %, which may not be the ideal values, but were chosen to demonstrate this exercise

In continuous processing, the size of the interface between batches can be minimized using experimentally measured RTDs. Since the RTD represents the pulse response of the system, it can be applied to represent the point response from a feedstock lot change, which behaves exactly like a tracer step change. For example, given the RTD measured from a continuous blender shown in Fig. 9a, the cumulative distribution function (CDF), *F*(*t*), shown in Fig. 9b can be derived. The CDF represents the fraction of new feedstock that will exit in the outlet stream as a function of time. For instance, the value is 0 at *t* = 0, meaning none of the new feedstock will be exiting. When the value of the CDF becomes 1, the old feedstock has completely exited and only the new feedstock would be exiting. The old feedstock would follow the inverse washout profile, represented by:

16 $$ W(t)=1-F(t) $$

As an example, batch boundaries were defined using 0.5 and 99.5 %, which are shown by the vertical lines in Fig. 9a, b. At a time of 30 s, the new feedstock would start to appear at the outlet of the system. At 160 s, the last of the old feedstock has left the system and the outlet only contains the new feedstock. Therefore, the material exiting from 30 to 160 s could be discarded as the transition interface (or released as a separate “batch,” to be recalled if necessary). The material before and after this time interval becomes two different and separate batches. The result is a short 130-s interface. At a total processing throughput for a formulation of 30 kg/h, the discarded interface would amount to about 1 kg of material. This is modest compared to the often used procedure of discarding the first and last portions of large batch-processed batches.

## Results

### Identifying Sources of Disturbances

A quality risk management process should include the assessment, control, communication, decisions, and review of risks to the quality of the drug product across the product life cycle. [38] In the work presented here, the focus is specifically on the first two parts, assessment and control, as they relate to content uniformity. A risk assessment includes identifying hazards, estimating the risk, and evaluation. Although there are an infinite number of hazards that can occur in any process, any unmonitored risk is based on both the probability and severity of the hazards. However, adequate detection and process controls can be utilized to reduce or eliminate risks.

In a continuous direct compaction line, the highest probability for content uniformity risk is at the feeders and blender. Assuming the blend is uniform at the exit of the blender, there is a very low risk of content uniformity issues arising. A properly designed continuous blender should have no dead zones and should have enough radial mixing to blend multiple components into a uniform mixture. Typically, the real issue is not the blender, but instead the composition of the inlet stream. If the ingredients in the inlet streams are not entering the blender at the correct ratios, no amount of blending will correct the composition of the blend. The feeders and the downspout from the feeders are the most likely cause of content uniformity risks.

**Fig. 10:**
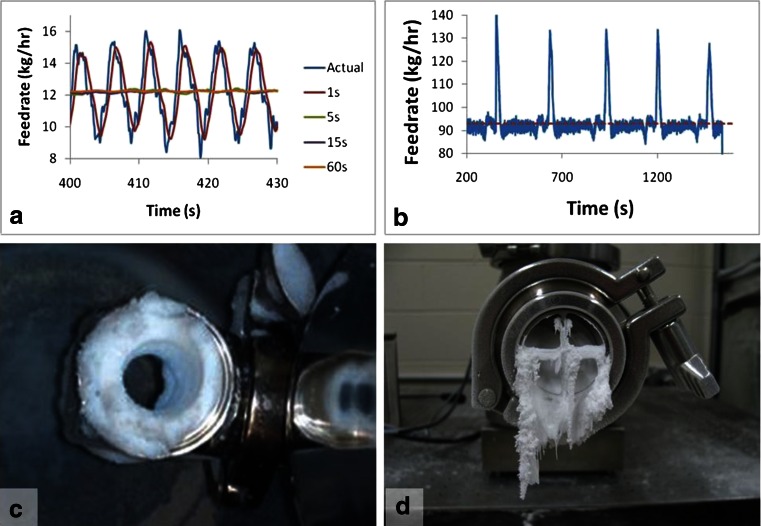
Sources of content uniformity variability: **a** feeder fluctuations, **b** deviations caused by refill, **c** downspout accumulation, **d** feeder bearding

The recommended feeders for pharmaceutical continuous processing are loss-in-weight feeders, which use internal gravimetric control based on load cell measurements. Gravimetric control greatly reduces the risk of feeder error. However, a few hazards that may arise have been identified as the following:

- Poor load cell calibration can cause the feeder to dispense at the wrong rate with the feeder’s controller unable to detect an issue. This is an operator error that will require system shutdown to correct. Detection depends on downstream PAT or monitoring the feeder’s drive speed. A calibration problem may be indicated by significant deviation from the historic behavior of the feeder’s screw speed while the reported load cell measurements remain within range.
- Some feed rate fluctuation (see Fig. 10a) is unavoidable. Fluctuation can be minimized with proper design, but still poses a potential risk.
- Disturbances can lead to deviations (i.e., hopper refill). See Fig. 10b. The most common cause of significant deviations in the feed rate of the feed stream is caused during hopper refill. When refilling, the feeders temporarily operate in volumetric mode and therefore do not correct for the density changes associated with hopper refill. This can be minimized with refill scheduling optimization, but still needs to be considered a potential risk [39].
- Downspout accumulation (see Fig. 10c) can cause a sudden rise in concentration of a component if accumulated material suddenly breaks off and falls. This typically indicates a design problem and requires redesign. However, small accumulation may still occur.
- Feeder bearding (see Fig. 10d) can also pose a risk when the material suddenly discharges and falls.

This above list of common feeding hazards is not exhaustive. Depending on the formulation and process, there may be other unlisted hazards, or the ones listed here may not be relevant. The cases displayed in Fig. 10 are all extreme cases and will not necessarily occur to the same degree with every powder. These can be summarized into two different cases that require analysis: fluctuations and pulse disturbances.

### Feeder Fluctuations and Filterability of the Mixer

Due to the intrinsic physics of powder flow, there will be some degree of variability in the feed stream. This variability can be minimized through feeder and tooling selection [40–42], but in any case, these fluctuations need to be quantified, and the system needs to be designed to handle these unavoidable variations. Typically, feeders would feed individual components into a continuous blender, blending them into a homogenous mixture through radial mixing. If the blender were a perfect plug flow mixer, then, the variations from the feeders will pass through the blender causing variations in content uniformity. Axial mixing within the blender enables a secondary function of smoothing or filtering out feeder variability. The degree to which this occurs depends on the residence time distribution of the blender and the magnitude and frequency of the fluctuations from the feeder.

The relations between the feeder and the blender can be evaluated using the Fourier series analysis demonstrated by Gao et al. [43] This paper defines the filterability, which quantifies a blender’s variance reduction ratio as a function of the frequencies of fluctuations. The filterability function can be derived from any residence time distribution. Similarly, the feed stream from a feeder can also be transformed into the frequency domain [40, 41].

**Fig. 11:**
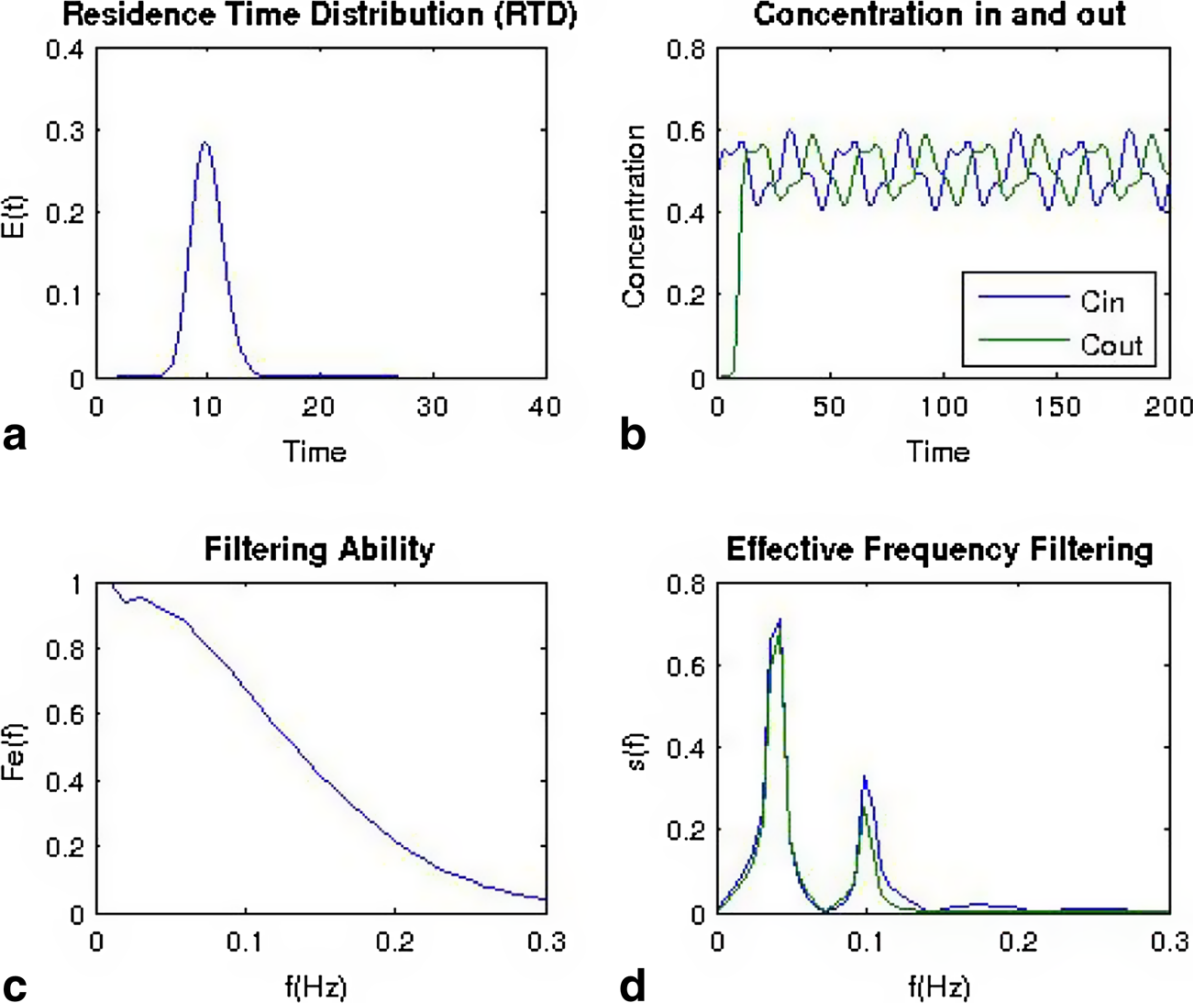
Simulated results for a bi-modal sine wave feed stream being fed to a blender with a narrow residence time distribution (in comparison to Fig. 12). **a** Residence time distribution. **b** Concentration profiles for the inlet and outlet of the blender. **c** Calculated filtering ability of the blender as a function of frequency. **d** Frequency domain of inlet and outlet streams

**Fig. 12:**
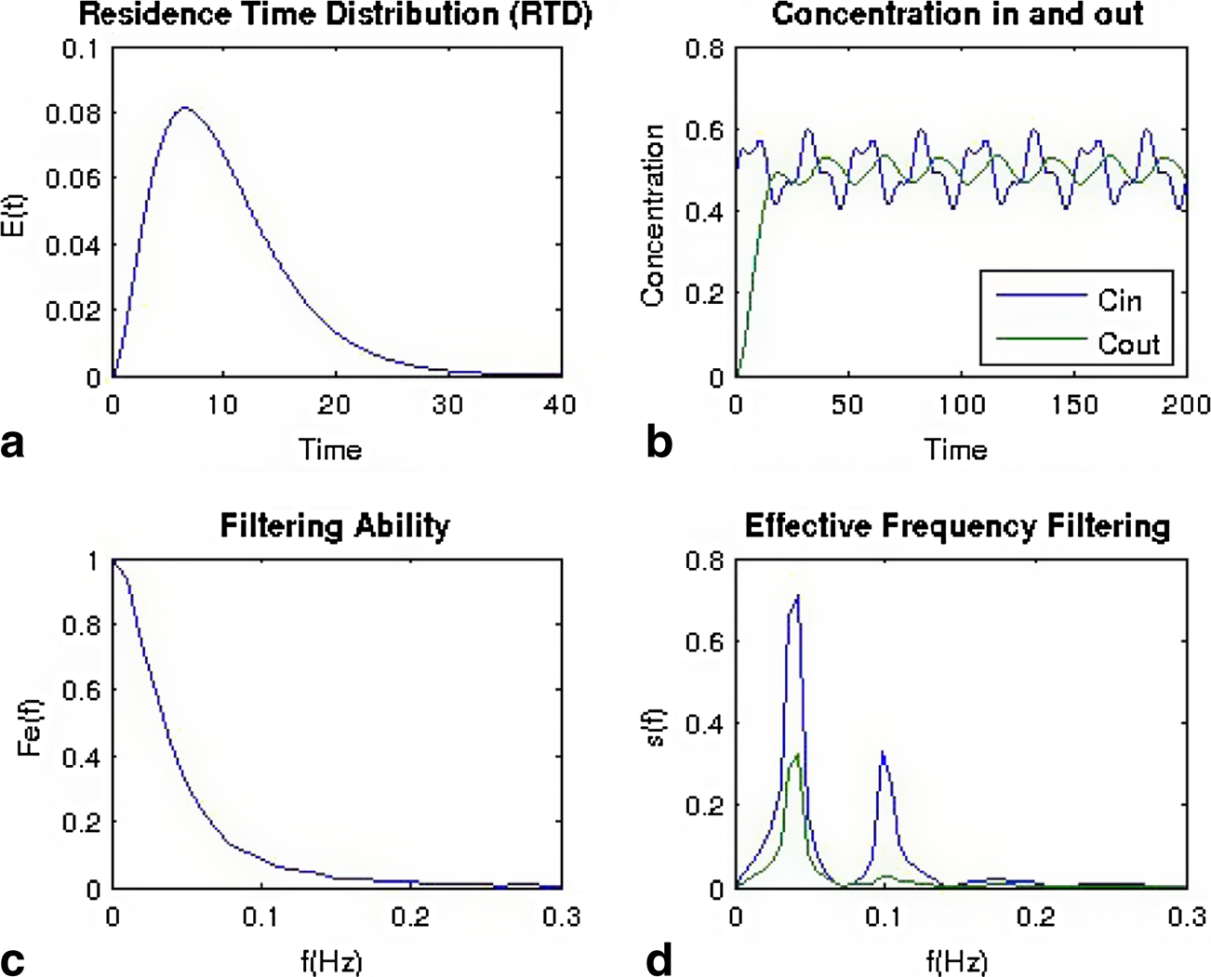
Simulated results for a bi-modal sine wave feed stream being fed to a blender with a broad residence time distribution (in comparison to Fig. 11). **a** Residence time distribution. **b** Concentration profiles for the inlet and outlet of the blender. **c** Calculated filtering ability of the blender as a function of frequency. **d** Frequency domain of inlet and outlet streams

The effect of residence time distribution on an incoming feed stream is shown in Figs. 11 and Fig. 12. Figure 11a shows a very narrow RTD, and Fig. 12a shows a broad distribution. Using the same feed stream, a bi-modal sine wave with frequencies of 0.05 and 0.1 z results in a very different behavior as shown in Figs. 11b and 12b. For the narrower distribution, the bi-modal sine wave is only shifted in the time scale, but the shape is nearly identical before and after the blender. For the broad distribution, which has significantly more back-mixing, the higher frequency is filtered out, and the amplitude of the lower frequency is reduced. These results are more clearly reflected in the frequency domain plots of Figs 11d and 12d.

For the filtering ability plots in Figs. 11c and 12c, a value of 1 indicates that fluctuations will pass through, and a value of 0 indicates that the fluctuation has been spread and therefore reduced in magnitude. Figure 11c shows the filtering ability for the narrow distribution, which will not filter out most fluctuations with frequencies longer than 0.15 Hz. In contrast, Fig. 12c shows the filtering ability for the broad distribution, which filters most fluctuations above 0.05 Hz.

**Fig. 13:**
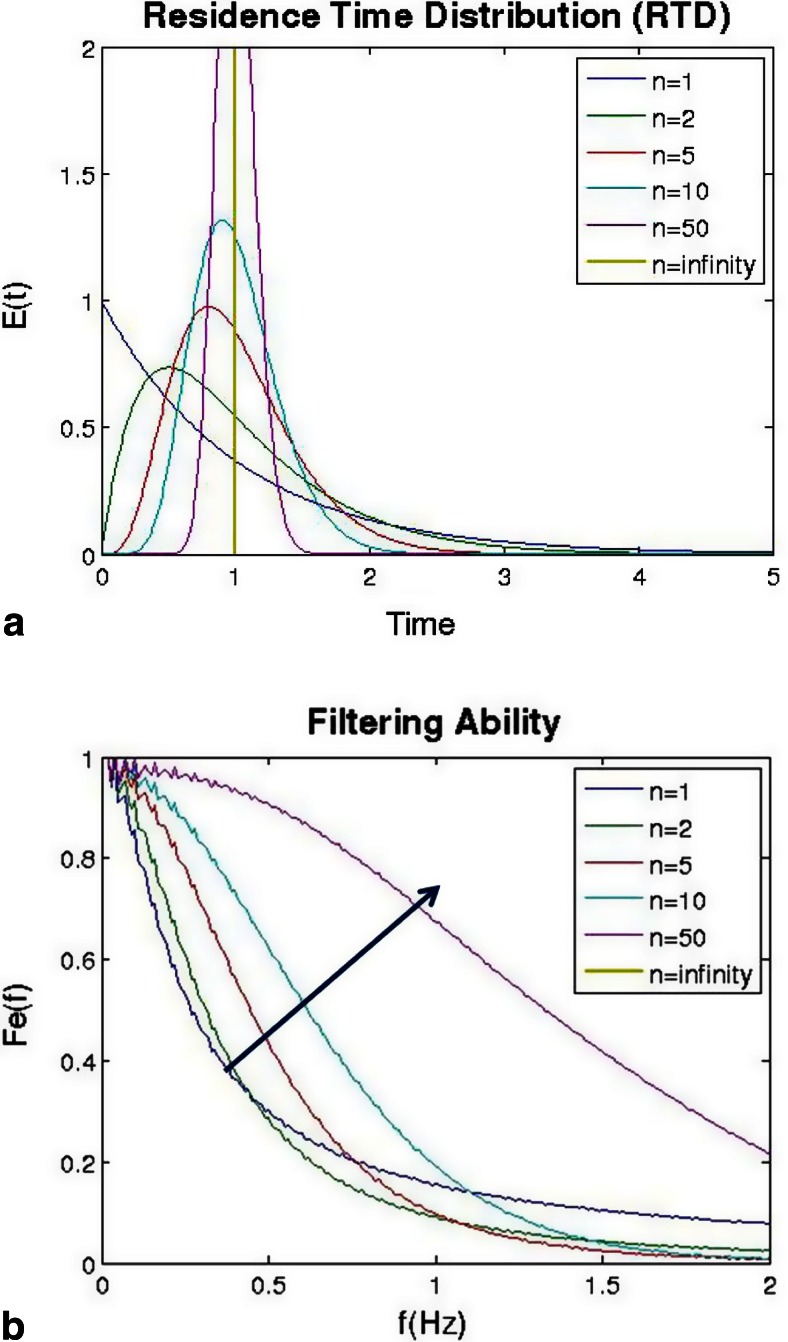
Effect of changing number of tanks in the tanks-in-series model: **a** residence time distribution and **b** ability to filter fluctuations of different frequencies

**Fig. 14:**
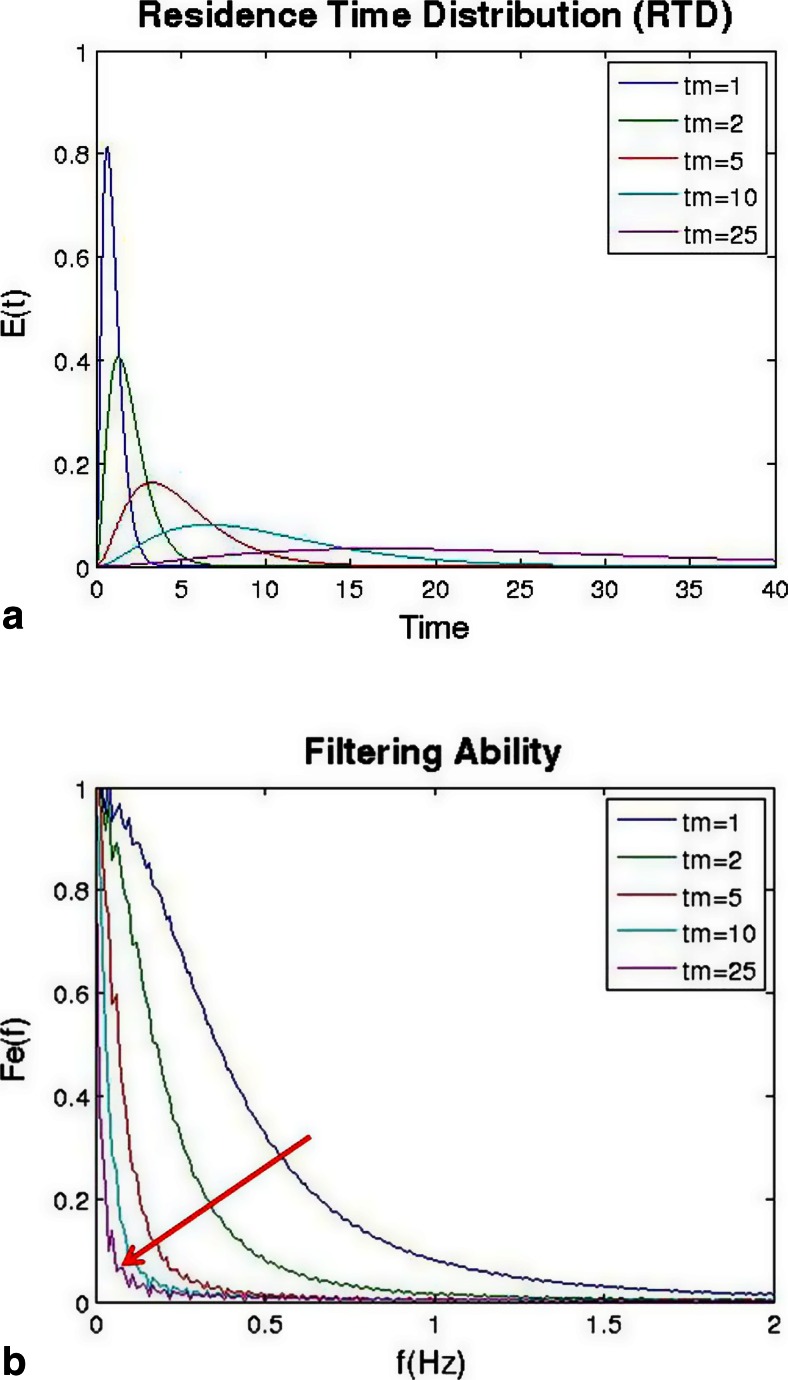
Effect of changing the mean residence time in the tanks-in-series model: **a** residence time distribution and **b** ability to filter fluctuations of different frequencies

The effect of changing the parameters of the tanks-in-series model is shown in Figs. 13 and 14. Figure 13a shows the residence time distributions as the number of tanks was increased from 1, which resembles a CSTR, up to infinity, which resembles that of a PFR. As the number of tanks was increased, the variance of the distribution decreases, which is indicated by a narrower distribution. Figure 13b shows that as the number of tanks increased, the ability to filter fluctuations decreased, which is indicated by the filterability increasing towards a value of 1.

Figure 14a shows the effect of increasing mean residence time on the shape of the residence time distribution. The mean residence time was increased from 1 to 25 s using the tanks-in-series model. Due to the arrangement of parameters within the equation of the model, an increase in mean residence time also increases the variance, which is shown by the broadening of the distribution. This resulted in a significant amount of back-mixing, which improved the ability to filter fluctuations as shown in Fig. 14b. Since the tanks-in-series model is a mono-modal distribution, filtering ability tends to decrease down to 0 with increasing frequency. This indicates that lower frequencies are more likely to pass through, whereas higher frequencies will be smoothed and filtered out entirely.

### Traceability of Pulse Disturbances

#### Simulated Pulse Disturbances

With the potential hazards identified, the next step of a quality risk assessment is risk analysis and evaluation. Hazards affecting content uniformity have a high potential to produce harm and need to be addressed. For most of the hazards identified above, the result is a sudden pulse-like addition of a component, which may cause a significant deviation from product content specification. Using the residence time distributions for each unit operation and the system as a whole, the significance of any pulse addition can be quantified via simulation.

**Fig. 15:**
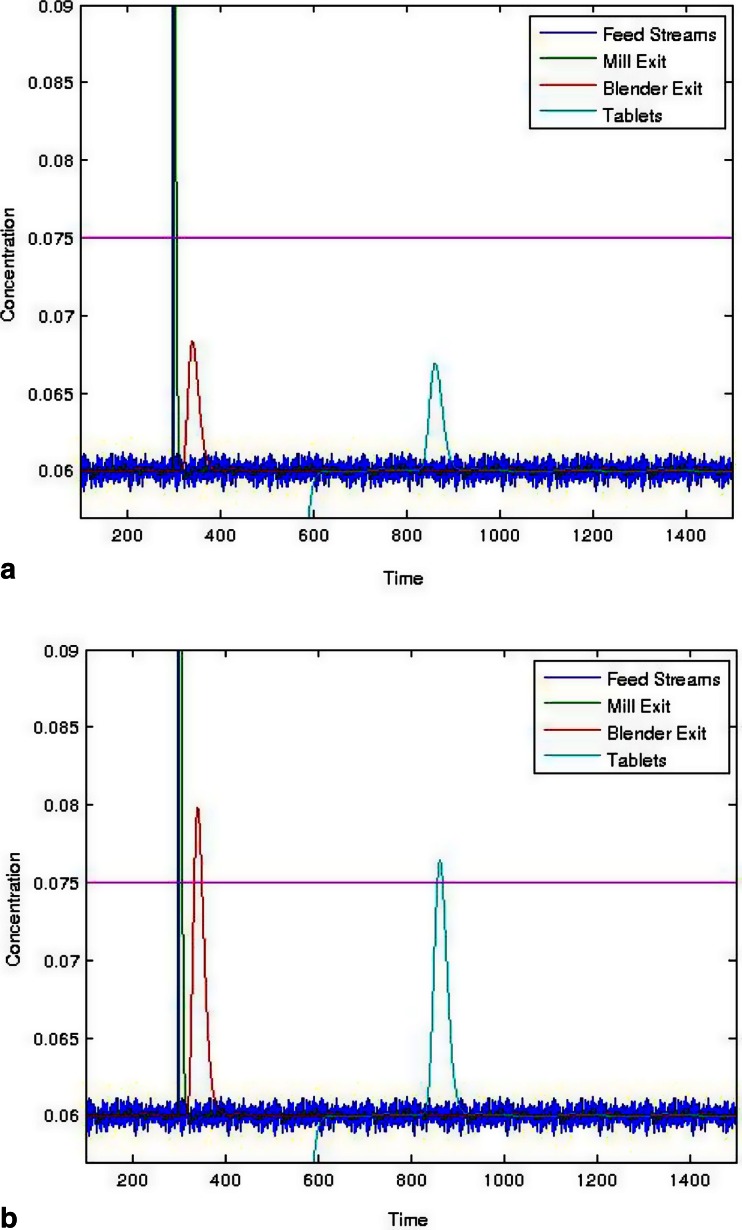
Simulation results showing the active pharmaceutical ingredient (API) concentration profile for the various unit ops and their response to a pulse of API added to the entrance to the mill. The blender has a mean residence time of 41.6 s and a standard deviation of 12 s. The sizes of the pulse are **a** 0.25 and **b** 1 g

**Fig. 16:**
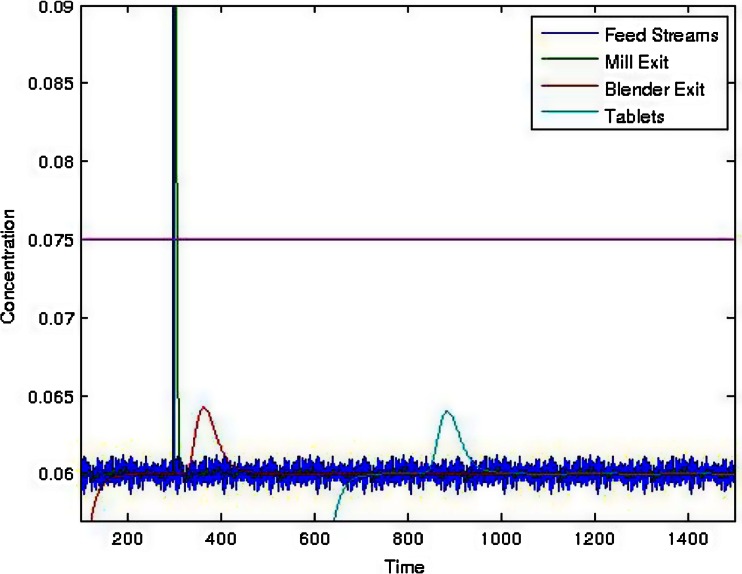
Simulation results showing the active pharmaceutical ingredient (API) concentration profile for the various unit ops and their response to a 1 g pulse of API added to the entrance to the mill. The blender has a mean residence time of 71.7 s and a standard deviation of 24.9 s

Consider a pulse input into the mill, such as from feeder bearding or downspout accumulation breaking off and falling. Figure 15a shows the response of a 0.25 g pulse into a direct compaction system with an overall throughput of 30 kg/h and a nominal active pharmaceutical ingredient (API) concentration of 6 %. The API pulse in the feed stream occurred at 300 s with the response from the mill immediately following. The spike in API concentration after passing through the blender occurred between 325 and 375 s, which added a significant amount of spreading due to back-mixing. Finally, the tablets exited between 800 and 900 s. This resulted in tablets within specification, <7.5 % (125 % of 6 %), meaning that no action was needed. However, if the pulse was increased to 1 g, as shown in Fig. 15b, there would be tablets out of specification (OOS). In this case, detection of the disturbance should trigger an exceptional event and corrective action should be taken, such as the removal of the OOS material from the product stream. Without predictive modeling, this would present a significant challenge. If material testing indicates a high probability of one of the identified hazards rather than a rare exceptional event, then, the system should be designed to handle that hazard, which is quality by design (QbD). Figure 16 shows the results for a system where the blade pattern in the continuous blender was changed, which caused it to have a broader residence time distribution. This resulted in a more robust system that could handle a 1 g pulse of the API without generating OOS product.

#### Sampling Frequency/Adequate PAT

Online process analytical technology is crucial for control of any continuous manufacturing process. However, its implementation is not as simple as adding sensors to measure properties of the blend at various stages in the system. Measurement needs to be meaningful, which requires a measurement that is representative, accurate, and timely. In batch manufacturing, the challenge is typically obtaining a measurement that is representative of the batch, because sensors or sampling is very localized. In continuous manufacturing, timely measurements are the larger challenge.

It is important to highlight a critical difference between a batch process and a continuous process. A batch process varies with time, whereas a continuous process varies primarily with respect to the spatial dimension. This means that the measurement at a fixed location in a batch process will be different at the beginning as opposed to the end. In a continuous process, this is not the case. If a sensor was fixed at the entrance to a continuous blender, the sensor would see the individual unmixed components throughout the entire processing time. If the sensor was fixed at the exit of the blender, the sensor would see a fully mixed blend after a short steady-state start-up time and until the line is shutdown. A sensor in a batch process only measures the final blend at the end of processing, whereas a continuous process conducts many measurements of small sections of the final blend throughout processing. Therefore, the measurements from the continuous process are more representative of the entire product stream (and therefore of entire batches).

In a continuous system, the most meaningful measurement is to characterize the intensity and frequency of fluctuations in the process stream. This means the sensors must be fast enough to detect any of these disturbances, ensuring nothing important passes the sensor undetected. This would be equivalent to a high concentration pocket or a segregated section not being detected in a batch process, due to the section not being within a sampling region. To ensure that this does not occur in continuous processing requires investigating how a fluctuation would spread in the process. The most difficult fluctuations to detect in a feed stream are narrow pulses, but as they progress along the system, pulses are spread based on the residence time distribution (RTD). Thus, the RTD contains the information needed to design the sensing system in order to ensure that pulse fluctuations do not travel through the system undetected.

**Fig. 17:**
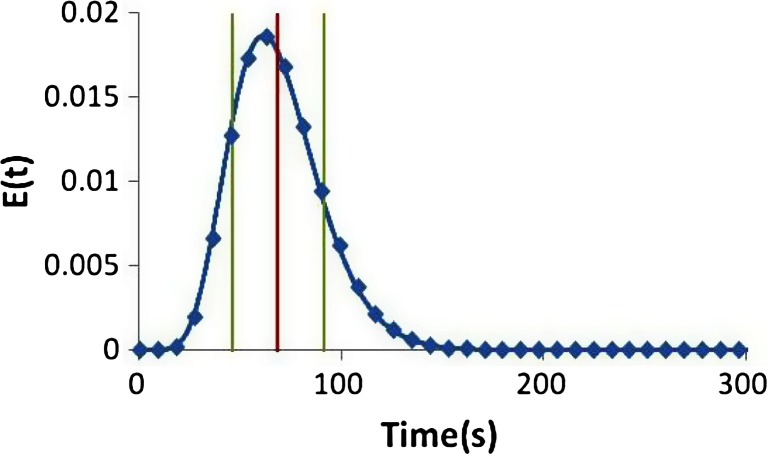
Residence time distribution with *vertical lines* representing the mean (68.8 s in *red*) and standard deviation (22.4 s in *green*). The sampling interval represented by the *diamonds* is 8.96 s, which was selected based on using five points across double the standard deviation

Figure 17 shows an example residence time distribution from the continuous blender. In this plot, the mean (68.8 s) is represented with a single vertical red line, and the standard deviation (22.4 s) is represented with two vertical green lines spaced on either side of the mean by the value of the standard deviation. It is logical to assume detection of the downstream response is easier than detecting the pulse disturbance itself. If the system were “plug flow,” the perturbation would be largely unchanged as it travels along the system, meaning that a pulse into the system would result in a pulse response. This would be difficult to detect without a very rapid measurement. Fortunately, this is not the case, and the sampling frequency only needs to be fast enough to catch a disturbance equivalent in shape to the RTD. A reasonable approach is to use a sampling or measurement regime that results in three to five measurements across the time interval represented by double the width of the RTD, which is quantified by its standard deviation. The following equations can then be used to define the maximum time between sampling and the minimum sampling frequency:

17 $$ {\tau}_{\mathrm{sampling}}=\frac{2\sigma }{n_{\mathrm{samples}}} $$

18 $$ {f}_{\mathrm{sampling}}=\frac{1}{\tau_{\mathrm{sampling}}}=\frac{n_{\mathrm{samples}}}{2\sigma } $$

where *n*_samples_ represents the number of samples and *σ* is the standard deviation of the RTD. For the RTD represented in Fig. 17, this would result in a sampling time of 8.96 to 14.93 s or a sampling frequency of 0.07 to 0.11 Hz. Utilizing high-frequency PAT sensors as defined by Eq. (18) would ensure adequate sensing to determine the approximated shape of the RTD. Aided by a simple peak detection algorithm, most significant spikes can be easily detected.

**Fig. 18:**
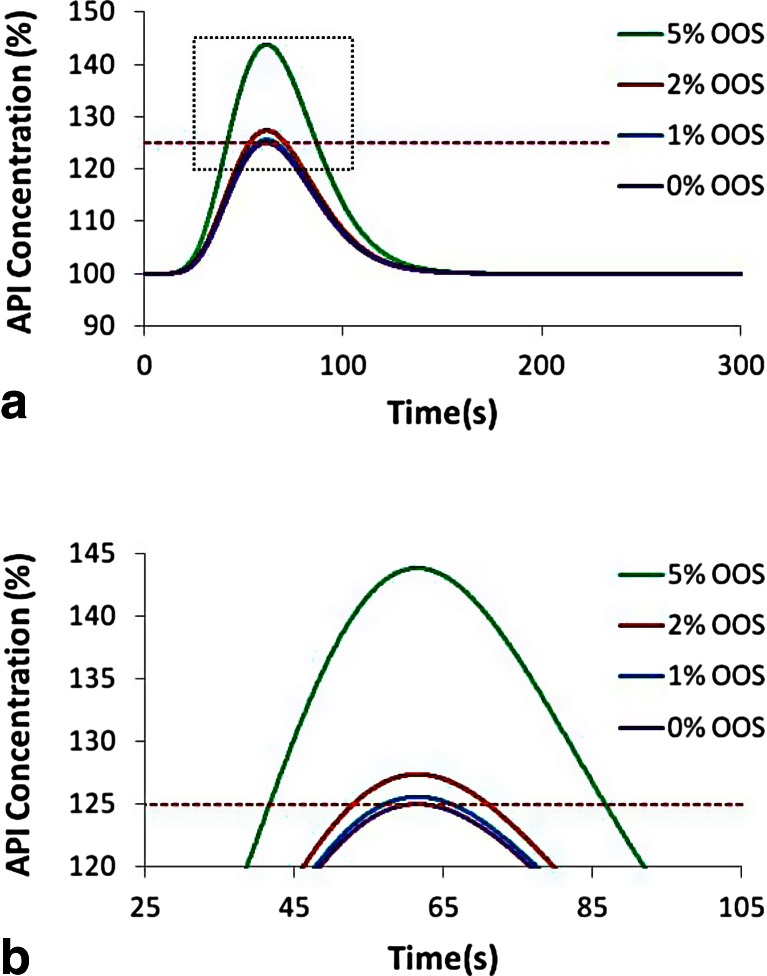
**a** API concentration pulse response resulting in various amounts of OOS material with a pass/fail value of 125 % API concentration. **b** Zoomed version for better resolution of the peak

However, using 125 % concentration as an upper limit for detection with a binary “pass/fail” outcome may result in smaller anomalies passing the PAT system undetected, resulting in small amounts of super-potent product, unless the sampling frequency is extremely high. Figure 18 shows the pulse response to various size pulses that result in differing amounts of super-potent product. The percentages for out of specification (OOS) product were calculated based on an assumed “batch” size based on a single disturbance event occurring once per 15 min (900 s) of continuous processing. Large deviations such as the one that results in 5 % OOS product, as shown in Fig. 18, will be detected easily as there will be several measurements indicating OOS material. However, the smaller deviations, 1 and 2 % OOS, do not exceed 125 % API concentration by much nor for very long, making online detection a challenge.

**Figure 19:**
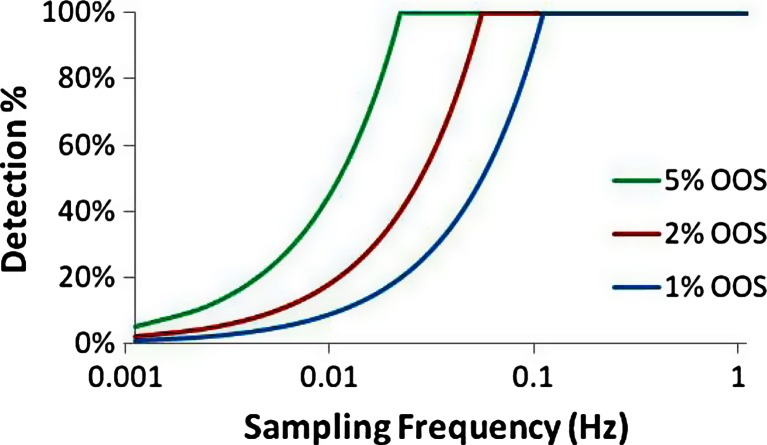
Probability of detection as a function of sampling frequency for pulses resulting in various amounts of OOS material: 1, 2, and 5 %

Figure 19 shows the percent chance of detection of a single pulse disturbance of various magnitudes, resulting in 1, 2, and 5 % OOS product, as a function of increasing sampling rate. The percent chance of detection is represented by the following equation:

19 $$ {D}_{\mathrm{continuous}}={t}_{\mathrm{batch}}{f}_{\mathrm{sampling}}P={n}_{\mathrm{samples}}P $$

where *P* represents the percent of material that is over the upper limit, *f*_sampling_ is the sampling frequency, and *t*_batch_ is the total time per batch (15 min = 900 s). With increasing sampling frequency, the probability of detecting a single exceptional event increases and eventually reaches 100 % for all three cases. Since the larger deviations are easier to detect, the detection percent is highest for 5 % OOS at any sampling frequency, which is followed by 2 % OOS, and finally 1 % OOS. The chance of detection reaches 100 % at the following sampling frequencies (and sampling time intervals) for the various deviations: 0.022 Hz (45 s) for 5 % OOS, 0.056 Hz (18 s) for 2 % OOS, and 0.111 Hz (9 s) for 1 % OOS. This means that at any of these sampling rates, there is 100 % coverage for deviations of that respective size. However, as the percent of OOS material decreases closer to 0 % the ability to detect these very small deviations requires infinitely faster sensing.

**Fig. 20:**
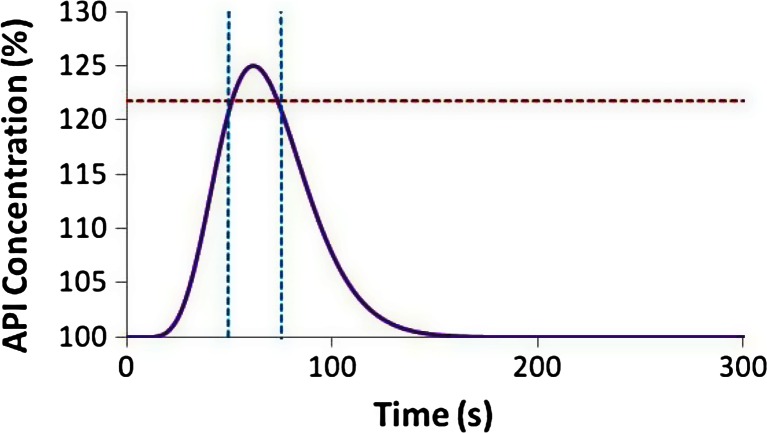
Concentration profile for a pulse response resulting in a peak of 125 % concentration. The *red horizontal dotted line* indicates a 121.75 % limit and the two *vertical blue dotted lines* indicate the width of the standard deviation (22.4 s) of the corresponding RTD, which is shown in Fig. 17

**Fig. 21:**
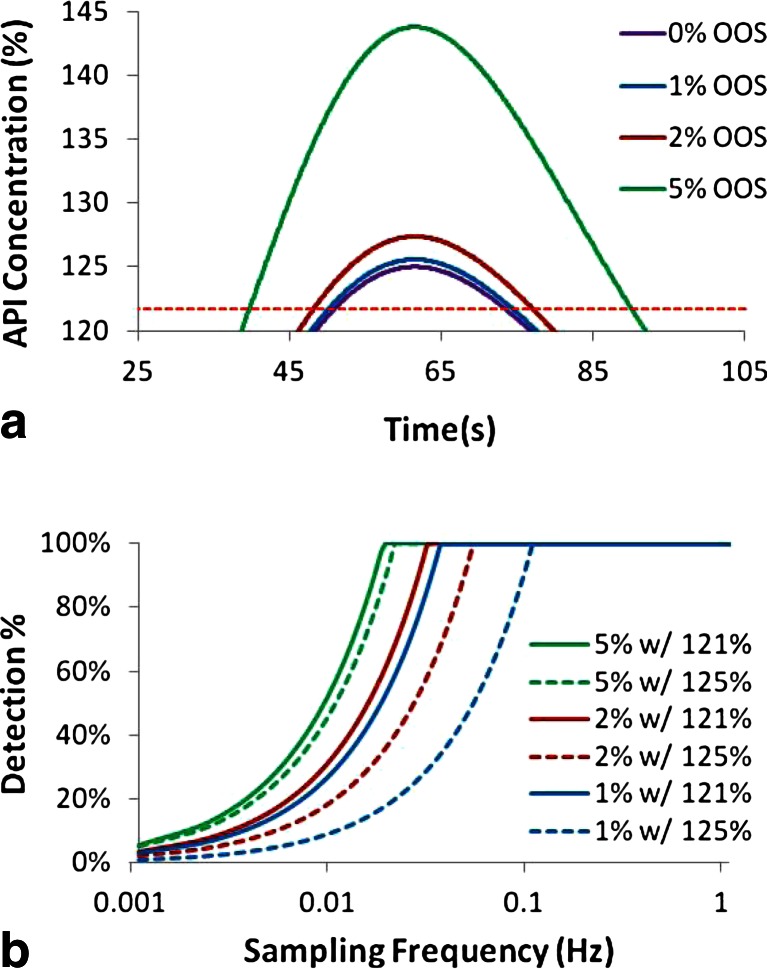
**a** API concentration pulse response resulting in various amounts of OOS material with a pass/fail value of 121.75 % API concentration. **b** Probability of detection as a function of sampling frequency for pulses resulting in various amounts of OOS material: 1, 2, and 5 % for both 121.75 % limit and 125 % limit. OOS material is determined by 125 % limit in both cases

To increase the ability of slower or less accurate PAT sensors to detect OOS material, the upper limit for the binary pass/fail criteria should be lowered. To detect a deviation at even the smallest deviation above 125 % requires sensing the limit where a single point reaches 125 %. Figure 20 shows this limiting concentration profile that peaks at 125 % API concentration and shares the shape of the RTD displayed in Fig. 17. Lowering the upper limit to 121.75 % results in material that exceeds the limit for 22.4 s, which is also the standard deviation of the RTD. Assuming a sampling frequency as defined by Eq. (18) would ensure that a few measurements are made during this interval allowing for adequate detection. Figure 21a shows a depiction of the material that would fail if the upper limit were reduced to 121.75 %, and Fig. 21b shows the corresponding chance of detection plotted as a function of increasing sampling frequency. The chance of detection reaches 100 % at the following sampling frequencies (and sampling time intervals) for the various deviations: 0.02 Hz (50 s) for 5 % OOS, 0.036 Hz (28 s) for 2 % OOS, and 0.042 Hz (24 s) for 1 % OOS. For comparison, the similar plots are also shown as dotted lines for the case using 125 % as the upper limit. For the smaller deviations, 1 and 2 % OOS, the improved detection ability is dramatic, whereas the larger deviation, 5 %, has less improvement.

**Fig. 22:**
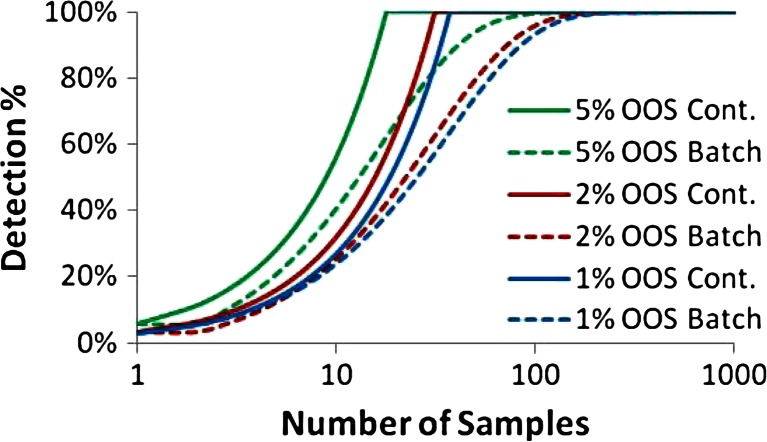
Probability of detection as a function of sampling frequency for pulses resulting in various amounts of OOS material: 1, 2, and 5 % for both a continuous process with online PAT (*solid lines*) and a batch process (*dotted lines*) with off-line random sampling. OOS material is specified by an upper limit of 125 % concentration, and the limit used for detection is 121.75 % concentration

The advantage of the continuous measurements of PAT versus sampling of a batch after processing is shown in Fig. 22. The sampling frequency for the continuous PAT measurements were translated into number of samples based on an assumed 15 min of processing for a batch allowing for direct comparison to a batch with a similar amount of OOS material. Differing from the continuous case which utilizes PAT, the batch curve assumes the sampling is completely random:

20 $$ {D}_{\mathrm{batch}}=1-{\left(1-P\right)}^{n_{\mathrm{samples}}} $$

PAT sensors in a continuous system have a set sampling frequency, which ensures that each measurement is observing a different section of material. Therefore, the sampling coverage and ability to detect all deviations will rapidly approach 100 %, at which point no deviation will pass the sensors undetected. To reach this same amount of coverage, a completely random or batch process will require orders of magnitude more samples. An initial comparison of continuous versus batch processing made from this plot is that there will be more correctly failed batches for a continuous process. However, this is not entirely the case, as these PAT sensor measurements allow downstream batch correction, such as a rejection chute, ensuring batches that would have failed do not contain any OOS material and therefore are of higher quality than batch processing with random sampling could ever achieve.

## Conclusions

Methods were presented to address challenges of batch definition, raw material traceability, and adequate PAT sensor frequency as it pertains to continuous manufacturing with reference to regulatory requirements. At the present time, available ICH guidances offer little explanation on implementation for continuous systems. Although batch definition is left open for the manufacturer to specify, other requirements, such as recording specific identification for each component within the batch records, make production changes, such as a feedstock lot change, a favorable factor for specification. To minimize crossover between batches, it was suggested to measure residence time distribution to quantify and define reasonable boundaries to remove the interface between batches, which may contain multiple batches of components.

To access and control risks associated with content uniformity, higher probability hazards were identified, categorized, and discussed. Solutions to these potential risks were presented where raw material traceability was a prevalent focus and a significant part of the solution. Residence time distribution (RTD) play an important role in raw material traceability as it characterizes the spreading of the materials through the system. Thus, a disturbance could be predictively tracked through the entire continuous system, allowing for downstream control or even removal of the affected material. Coupled with a diagnostic system, corrective action at the onset of a disturbance is possible (i.e., fault mitigation).

An important requirement of any PAT instrumentation is the reliability of the measurements, which includes a sensing frequency high enough to detect all significant disturbances. Since pulse disturbances would require an extremely fast sensor for detection, it was suggested that a downstream sensor could be used. This would not require such high-frequency sensing, but instead would only need sensing fast enough to detect the downstream response, which would have the shape of the RTD. This resolves the potential issue of OOS material passing through to the product undetected and also sets up some of the conditions needed for real-time release testing (RTRt). RTRt also requires verification that the measurements from PAT instrumentation reflect the testing results that would be collected in traditional batch release testing.

Although the methods described focus on direct compaction, they apply to any continuous processing system. To apply these methods to other continuous formulating techniques requires only minor changes. Together, the methods presented in this work bring continuous processing in the pharmaceutical industry to the point of understanding for actual commercial installations.
